# Supplementary figures and images for: Chronobiologically-informed features from CGM data provide unique information for XGBoost prediction of longer-term glycemic dysregulation in 8,000 individuals with type-2 diabetes
Source: PLOS Digit Health. 2025 Apr 9;4(4):e0000815. doi: 10.1371/journal.pdig.0000815 (PMC11981153; doi:10.1371/journal.pdig.0000815)

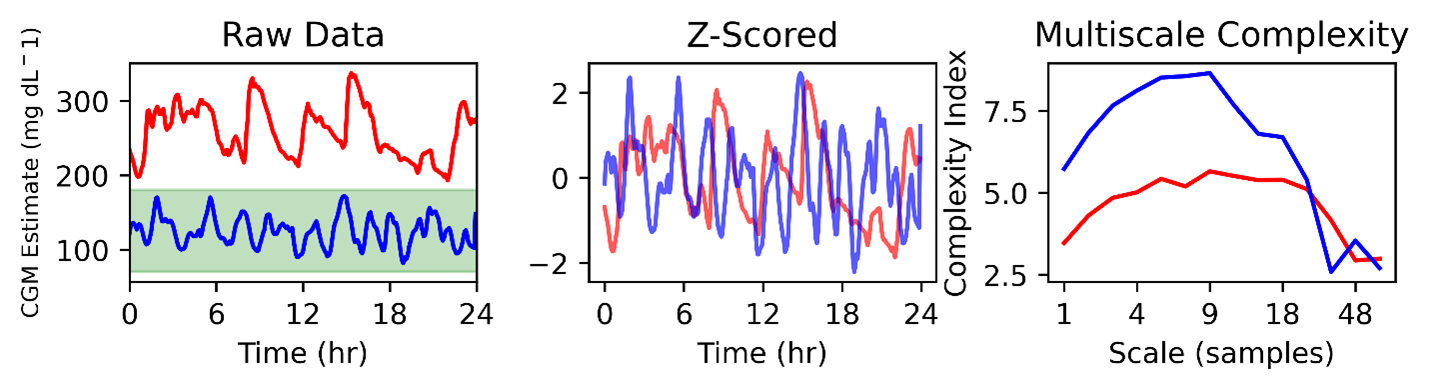

Supplement: S1 Fig — (TIF) [file pdig.0000815.s001.tif]

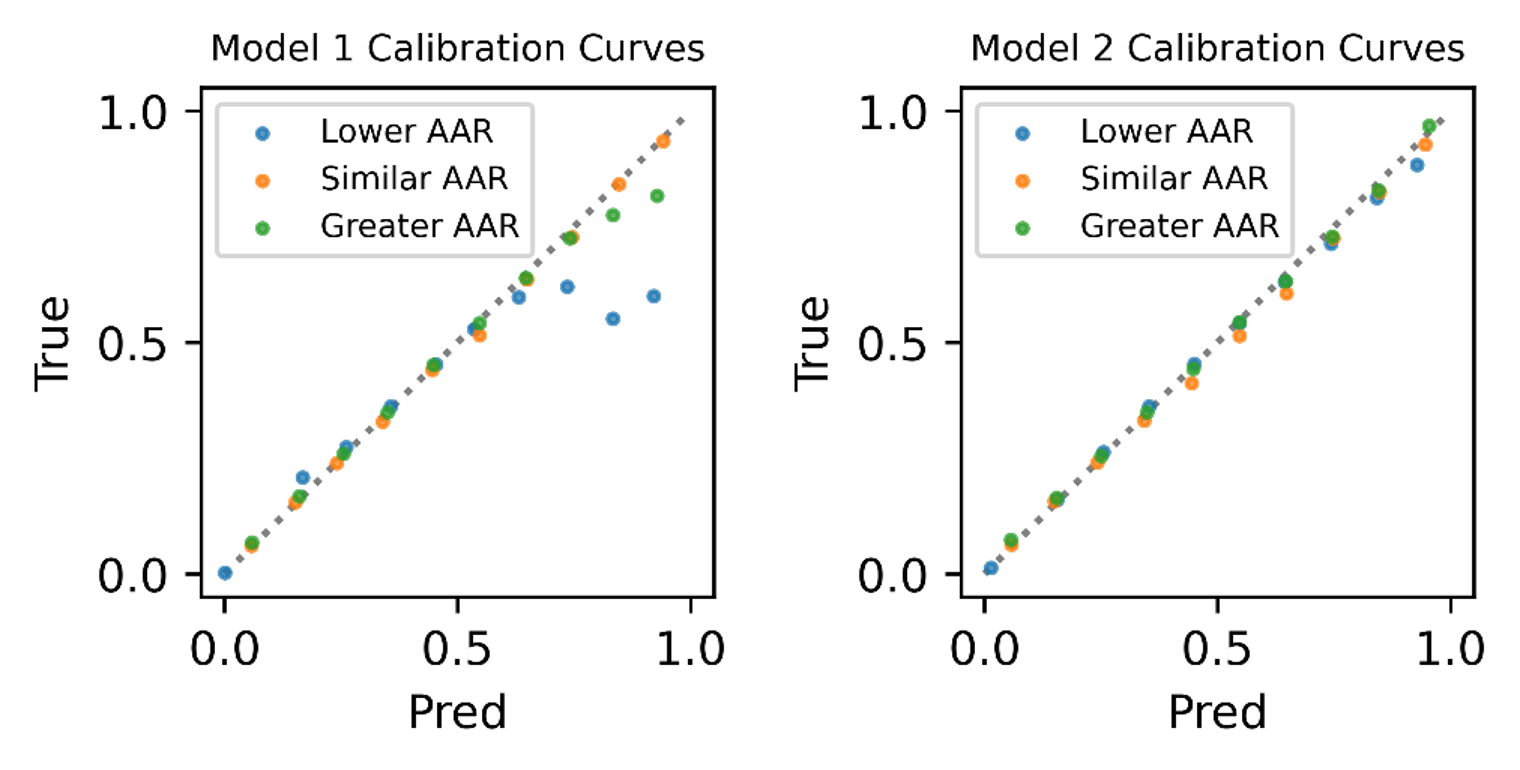

Supplement: S2 Fig — (TIF) [file pdig.0000815.s002.tif]
