# Supplementary material for: Chronobiologically-informed features from CGM data provide unique information for XGBoost prediction of longer-term glycemic dysregulation in 8,000 individuals with type-2 diabetes
Source: PLOS Digit Health. 2025 Apr 9;4(4):e0000815. doi: 10.1371/journal.pdig.0000815 (PMC11981153; doi:10.1371/journal.pdig.0000815)
Supplement: Attachment — Submitted filename: Response to reviewers.docx [file pdig.0000815.s007.docx]

HALICIOĞLU DATA SCIENCE INSTITUTE

9500 GILMAN DRIVE

LA JOLLA, CALIFORNIA

92093-0555

BENJAMIN L. SMARR

ASST. PROF. BIOENGINEERING AND HDSI

December 12, 2024

Dear Dr. Leo Anthony Celi,

Thank you for providing review comments for our manuscript *Chronobiologically-Informed Features from CGM Data Provide Unique Information for XGBoost Prediction of Longer-Term Glycemic Dysregulation in 8,000 Individuals with Type-2 Diabetes*. We appreciate the thoughtful comments by the reviewers, and the opportunity to add clarity to the places they identify as in need. We have amended our manuscript to reflect the interests of the reviewers and are happy to provide the improved manuscript for your consideration.

We provide responses item by item below with line numbers as present in the ‘Revised Article with Changes Highlighted’ file.

*Additional Editor Comments*:

E.a Please provide separate figure files in .tif or .eps format only and remove any figures embedded in your manuscript file. Please also ensure that all files are under our size limit of 10MB. For more information about figure files please see our guidelines:
[https://urldefense.com/v3/__https://journals.plos.org/digitalhealth/s/figures__;!!Mih3wA!GKl2g9YyZ9EBLqG6ug-DV2Edr0I3AVgixfbpWMKyMSbHUZm_BsvfsQ8vAnSdf3Vd2TqqLEVhG3RTohs$](https://urldefense.com/v3/__https:/journals.plos.org/digitalhealth/s/figures__;!!Mih3wA!GKl2g9YyZ9EBLqG6ug-DV2Edr0I3AVgixfbpWMKyMSbHUZm_BsvfsQ8vAnSdf3Vd2TqqLEVhG3RTohs$)
[https://urldefense.com/v3/__https://journals.plos.org/digitalhealth/s/figures*loc-file-requirements__;Iw!!Mih3wA!GKl2g9YyZ9EBLqG6ug-DV2Edr0I3AVgixfbpWMKyMSbHUZm_BsvfsQ8vAnSdf3Vd2TqqLEVhYErgMvM$](https://urldefense.com/v3/__https:/journals.plos.org/digitalhealth/s/figures*loc-file-requirements__;Iw!!Mih3wA!GKl2g9YyZ9EBLqG6ug-DV2Edr0I3AVgixfbpWMKyMSbHUZm_BsvfsQ8vAnSdf3Vd2TqqLEVhYErgMvM$)

*For this updated submission, the figures have been provided as separate .tif files and have been removed from the manuscript file.*

E.b The manuscript does not comply with PLOS's data availability policy. You must make all underlying data fully available without restriction, either as part of the manuscript or deposited in a public repository. Please address this issue promptly.

*Data will be deposited in the University of California, San Diego public repository with the associated DOI of the manuscript following potential acceptance (line 299-300).*

E.c The reviewer was uncertain about the rigor of the statistical analysis. Ensure that all statistical methods are clearly described and validated, and consider including a detailed explanation of your analysis approach.

*We have gone through the manuscript and made sure we are clear with our descriptions of which statistical tests are used to support which claims (lines 64-66, 74-77, 103-104, 108, 115, 116).*

E.d The manuscript needs improvements in language clarity and presentation. There are typographical errors, unclear explanations, and a need for better structure in presenting methodology and results.

*Thank you for the feedback. We have updated the manuscript to hopefully improve clarity.*

E.e The current validation approach using a train-test split is a good starting point but insufficient. I recommend incorporating cross-validation methods to enhance the robustness of your model's performance. Additionally, external validation using independent datasets would strengthen the generalizability of your results.

*We thank the Editor for their recommendation. We have performed 5-fold cross-validation and have added the performance metrics across all folds as a table in the supplements, and have included it in the methods section (line 289).*

E.f While the study emphasizes chronobiologically-informed features, a deeper exploration into their interpretability and clinical relevance is needed. Discuss how these features can translate into actionable insights for patients and clinicians, providing examples or case studies to illustrate their practical implications.

*We have added more context to the Discussion outlining the interpretability of the chronobiological features. Specifically, we call out that time-of-day standard deviation may be related to eating habits. While the data used for this work cannot be evaluated for causality between the time-of-day standard deviation and time-in-range, there exists a relationship between better regulation the next day and lower variance across days. We also cite prior work that describes how estimates of complexity may indicate differences in the regulation of glucose at smaller timescales, which may be a better indicator as a risk factor instead of something to be fixed – as controlling the 5-min fluctuations of one’s glucose levels without insulin is intractable. (lines 147-155, 169-173).*

E.g Include a more detailed discussion on the selection process for the chronobiological features and their relative importance in the model. A comprehensive analysis of which features contribute most to the predictive power of the XGBoost model would be beneficial.

*We have added more context to the Discussion outlining the impact of the features that contribute most to the predictive power and post-hoc analysis as to why they may be the most impactful (lines 167-171).*

E.h Acknowledge and address potential biases related to the CGM data provided by Dexcom. Discuss any inherent biases or limitations of the dataset and their potential impact on the generalizability of your findings.

*We have included biases related to how the underlying populations that use Dexcom devices may not be equivalent to populations that use different CGM devices (lines 189-196).*

E.i Provide a comparative analysis with other state-of-the-art methods or models for predicting long-term glycemic dysregulation. This will help contextualize the performance gains achieved with your approach.

*We appreciate this feedback. The goal of the manuscript was to highlight the benefits of chronobiologically-informed feature engineering. This was performed by means of extracting informative covariates about the target variable that are independent of the typical statistical features (central tendency, variance, skew, etc.) extracted from longitudinal timeseries. To this end, the XGBoost model was more of a validation of the addition of these novel features for the feasibility of predicting longer-term glycemic control. It was also selected for its built-in ability to handle nonlinearity and missing data. Within the scope of this work, we did not aim to maximize the performance for an “in production” ML algorithm. Therefore, we only used XGBoost since that was sufficient to test the hypothesis that this feature engineering rendered improvement. In the future, if a pure machine learning focused project were to be developed, hyperparameter optimization would be an ideal option for maximizing the performance of a model that may be placed in production. We have added a brief summary of this to the Discussion (lines 163-167, 175-181).*

E.j Figure 1:
- Clarify x-axis labels in panel D and provide a legend explaining quantile ranges.
- Include additional annotations in panels E and F.
- For panel G, provide more details on the von Mises distribution.

*Figure 1 has been updated per these recommendations. The quantile ranges have also been added to the figure caption.*

E.k Figure 2:
- Add grid lines in panel A for better trend visualization.
- Include trend lines or additional statistical summaries in panel B.
- Provide explanatory text or labels for key trends in panel C.
- Include information on statistical significance in panels D and E.

*Figure 2 has been updated per these recommendations.*

E.l Figure 3:
- Add more explicit labels or annotations in the left panel.
- Provide detailed statistical summaries or trend lines for gender-specific effects in the right panel.

*Figure 3 has been updated per these recommendations. The gender-specific effects have been removed for this version of the manuscript.*

E.m Figure 4:
- Summarize key performance metrics directly on the plots in panels A-C.
- Provide a brief discussion of impactful features in panel D and highlight key takeaways or additional annotations in panel E.

*The discussion section has been expanded to include key performance metrics and specific impactful features.*

E.n Table 1:
- Include percentages for gender distribution, age categories, and treatment types to enhance clarity.

*We have added percentages to Table 2 (the demographics table – originally Table 1) to enhance clarity (line 240).*

E.o Confirm that the data provided by Dexcom is de-identified in compliance with HIPAA regulations and other relevant privacy standards. Ensure that no identifiers are present, either directly or indirectly, that could lead to the identification of individual subjects.

*We have added the confirmation of data de-identification to the “Data Description” section of the Methods and have ensured that no identifiers are present that could lead to the identification of individuals subjects (lines 242-243).*

E.p Although the IRB waiver applies, mentioning its reference number is crucial. Please include this reference in your revised manuscript.

*We apologize for the confusion. We have reengaged the University of California, San Diego IRB for official confirmation of a waiver and will add the associated reference number upon a potential requested final version (line 235).*

E.q Discuss any potential ethical implications of the findings, particularly if the research could influence diabetes management practices or patient outcomes. Address how the findings will be communicated and used to ensure that they do not inadvertently harm or mislead patients.

*We have included a section in the Discussion related to the implications and interpretation of the findings. To summarize here, machine learning algorithms can historically reinforce biases due to the underlying data that is provided to them. The findings presented in this work are meant to convey that information about long-term stability can be extracted from CGM data (not only short-term deviations). As such, much more care should be taken into the interpretation of the results. Specifically, we recommend that these features be validated in a prospective study that incorporates patient eating habits, behaviors, and clinical endpoints to better understand if these features are identifying behavioral features, physiological features, or the combination of the two (lines 179-181, 214-217).*

***Reviewer #1:***

There are some comments regarding the submitted paper. However, the peer review process would be easier if manuscript lines were numbered to cite a certain line.

*Thank you for your comments. We have included line numbers in the revised version of the manuscript.*

**Major**
1.a How the authors justify for the ToDSD in the noon be the lowest and on the midnight be the highest. It’s known that the sensitivity for insulin decrease in the night and the glucose peaks at this time. Is there any evidence from the literature about this observation?

*We appreciate Reviewer 1’s comment on the lack of clarity/justification for the observation of minimum and maximum ToDSD at around noon and midnight, respectively. We have made the location of the statistical test (von Mises Criterion) and the figure it refers to (Figure 1G) clearer in the Results section (lines 73-78). We have also added more detail and references in the Discussion section that describe why the minimum and maximum ToDSD are at those times (lines 147-155).*

1.b The authors used certain time frames such as two weeks and one month as a sample of time from each patient. However, the authors didn’t mention how they chose that time for each patient. Is it the same time of the year for all patients or was it sampled randomly or more than one period were taken from each patient?

*We thank Reviewer 1 for pointing out the ambiguity in how the two-week and one-month samples were selected for each individual. We have added in clearer language in the* ***Methods*** *-* ***Variability of glucose concentrations at the same time of day (ToD) across multiple days reveals an increased risk of glycemic dysregulation*** *that the two-week windows were selected from the same time of year for all individuals based on the fact that all participants had CGM data during that time and had very little missing data per day (lines 255-256). We have also added a section in* ***Methods - Multi-scale complexity from longitudinal data identifies increased shorter-term variability in individuals with higher TIR*** *that the month-long window was selected from the same section of year for each individual based on similar criteria as the two-week selection (lines 279-280).*

1.c In Figure 3, there is no explanation or statistical evidence provided for the differences in mCI after controlling for the gender. The paper is presented as there is already a significant difference.

*We thank Reviewer 1 for pointing out the claim that age affects mCI differently between males and females. The reviewer is correct that this was not a significant comparison. As it was an accidental remnant of the exploratory data analysis phase, we have removed the claim from the section header and fixed Figure 3B accordingly (line 109).*

**Minor**
1.d The authors didn’t consider for the epidemiological factors in the study except for the age. These factors could include the ethnic groups and socioeconomic status for the patients included in this study. This should be stated at least as a limitation.

*We appreciate Reviewer 1’s comment regarding incorporating epidemiological factors, and agree that this would have strengthened the work. However, the data provided by the industry collaborator, Dexcom, did not include factors such as ethnic groups and socioeconomic status. We have added this as both a limitation and point for potential future work in the Discussion (lines 189-196).*

1.e To facilitate study repeatability, the authors should state how they performed calculations and processes on the data and using which software as they only included the machine learning part.

*We appreciate Reviewer 1’s point about repeatability. We have added a section at the end of the methods outlining which software and packages (and their respective versions) were used to perform the data analysis (lines 291-296)*

1.f The authors mentioned certain observations in the figures without further analysis and discussion. See e.g. the last sentence in the first result paragraph.

*We thank Reviewer 1 for identifying a location in the results where a p-value was forgotten (line 65). We have incorporated the p-value resulting from a Breusch-Pagan test for heteroskedasticity and have also included the linear fit of TIR data from ToDSD in Figure 1C.*

1.g The authors consistently present the results at the beginning of each paragraph, followed by a description of how they were measured. For example, in the statements "There is a negative correlation between ToD Standard Deviation (ToDSD) and time in range" and "The magnitude of ToDSD across multiple days is affected by ToD itself. In aggregate, subjects have a maximum and minimum ToDSD at around midnight and noon, respectively," as well as in other paragraphs. It would enhance clarity for the reader if the methodology or measurement process were discussed first, followed by the results. This approach would reduce potential confusion and make the findings more accessible. I recommend additional academic language editing to improve the overall readability of the paper and correct tenses.

*We appreciate Reviewer 1’s suggestion to include methodology/measurement process prior to the statement of results. We have modified the beginning of Results section 1 (lines 56-58) and Results section 2 (lines 80-82). We have also added more academic language throughout the updated manuscript to help with readability.*

1.h In Figure 1C, why does the range for average hours of TIR for patients span from 0-100? Was the data normalized?

*We thank Reviewer 1 for pointing out confusion in the y-axis label of Figure 1C. It has been corrected to reflect “Time in Range” as a percentage from 0% to 100%, which is the recommended method of reporting glycemic regulation per the American Diabetes Association.*

1.i Figure 3 consists of two charts, but they are not labeled with distinguishing letters or identifiers.

*We thank Reviewer 1 for pointing out the ambiguity in Figure 3 subplots. We have added labels ‘A’ and ‘B’ above their respective subplots and have pointed them out in the Figure 3 caption.*

1.j There is a spelling error in “glyc1emic” at the paragraph just before the last one in discussion.

*We thank Reviewer 1 for pointing out this spelling error. It has been corrected to ‘glycemic’ (line 219).*

***Reviewer #2:***

In this manuscript, the authors used Dexcom CGM data gathered from 8,000 T2D subjects to identify long-term structural features. They showed that adding these features in an explainable out-of-the-box XGBoost model improves the performance of predicting the severity of glycemic dysregulation compared to using time-series statistics alone. The idea behind the research is interesting, however, there are some point that should be considered:

2.a Why did the authors use only XGBoost model? They should use other ML algorithms and compare the results.

*We appreciate Reviewer 2’s question. The goal of the manuscript was to highlight the benefits of chronobiologically-informed feature engineering. This was performed by means of extracting informative covariates about the target variable that are independent of the typical statistical features (central tendency, variance, skew, etc.) extracted from longitudinal timeseries. To this end, the XGBoost model was more of a validation of the addition of these novel features for the feasibility of predicting longer-term glycemic control. It was also selected for its built-in ability to handle nonlinearity and missing data. Within the scope of this work, we did not aim to maximize the performance for an “in production” ML algorithm. Therefore, we only used XGBoost since that was sufficient to test the hypothesis that this feature engineering rendered improvement. In the future, if a pure machine learning focused project were to be developed, hyperparameter optimization would be an ideal option for maximizing the performance of a model that may be placed in production. We have added a brief summary of this to the Discussion (lines 163-167).*

2.b The parameters such as Accuracy, Precision, Recall, and F1-score should be mentioned.

*We thank Reviewer 2 for pointing out that the work would be strengthened by including information regarding other performance metrics. We have added a new table (Table 1) that includes the micro- and macro-level performances of area under the receiver operating characteristic curve (AUROC), precision, recall, F1-score, and accuracy (line 140).*

2.c In "Discussion", the authors should compare their results with the previously published papers and discuss about the advantages of their model.

*We agree with Reviewer 2 that results comparisons with previously published models would be ideal. However, to our knowledge, the data types and task are unique to this work and thus cannot be compared to prior work. Such prior work has largely focused on predicting/forecasting short-term hyperglycemic/hypoglycemic excursions, and many benchmark datasets and the associated models derived from them are well-documented. We have included a statement in the Discussion that outlines this reasoning (lines 175-181).*

2.d Overall, the manuscript has been written as a report, not a research paper.

*We appreciate Reviewer 2’s feedback regarding the style of the work. We have reordered some portions of the Results section so that it more clearly follows the hypothesis/methods/testing writing flow that may be easier for readers to follow.*

***Reviewer #3:***

Burks et al. investigated whether chronobiologically-informed features from continuous glucose monitoring (CGM) data has additional value on top of classical CGM features for prediction of glycemic regulation (longer term=next day). Some of these features and their association with classical CGM measures (TIR) are informative and intuitive (e.g. SD at different time points), while others are to a lesser extent (complexity index, see details below). In general, the study focuses on an interesting topic that is often overlooked, but some aspects could be strengthened, especially from a clinical perspective (there doesn’t seem to be a clinician among co-authors). The dataset is large for a CGM study and the statistical analysis was conducted with care, although the reporting could be more detailed (see details below). Neither data nor code are publicly available. I have the following questions/suggestions to further improve the manuscript:

**Major**
3.a Chronobiologically-informed features
As the authors described, most other studies used short-term features for prediction, so I really support extending this horizon. However, I don’t find the complexity index and its positive association with TIR clinically intuitive. CI (I suggest to avoid using this abbreviation, because 99% of readers with a medical background will confuse it with confidence interval), is based on the difference of consecutive measurements, so if someone has a completely flat curve in range, then it would be 0, if I understand correctly. At the same time, if someone has huge swings (which is also one way to have higher TIR), then CI would be expected to be higher. So for me it’s not intuitive how high complexity is associated with high TIR. Could the authors include a couple of examples (similar to Fig 1A) to demonstrate a couple of scenarios/profiles? I would like more arguments for looking at age- and sex-related associations (Fig 3), which I don’t find too relevant, but it might be because of the lack of intuition in general about CI.

*We appreciate Reviewer 3’s feedback regarding the utilization of the Complexity Index. The algorithm is performed on the standardized (Z-scored) sequence of data at each coursing scale (Methods). This preprocessing step is recommended by the creators of the Complexity Index since their original aim of the metric was to identify if different sequences had greater short term (point-to-point) fluctuations relative to the global variance of the data. The greater Complexity Index values in the CGM data for individuals with greater time in range reflects that short term fluctuations describe a greater proportion of global variance compared to individuals with less time in range. We have elaborated on this in the results section related to the Complexity Index to improve interpretability (lines 90-96) and have further included a visual outline of a high time in range and low time in range (and their respective mCI curves) in the supplements (Supplementary Figure 2). We have also chosen to continue with the acronym “CI” since it is in reference to the original method paper as well. We included the age-related associations to surface if the mCI curves were surfacing expected differences in complexity – that is, it has been identified that elderly individuals (across many physiological metrics) undergo “critical slowing down” which is a diminishing in fluctuation magnitudes or an inability to quickly return to baseline. We have added in a citation in the discussion and elaborated further on mCI in age as well (lines 204-206).*

3.b Choice of outcome/target
I’d like the authors to justify why this is the relevant outcome to predict. If a person had really good control (e.g. close to 100% TIR), then there is only one way to go from there: towards a higher area above range (AAR). The other way around is also true, with high values for the last two days, one is more likely to improve, which is the well-described phenomenon of regression to the mean. This is also reflected in what the top 3 strongest predictors are. If the authors argue for using this outcome, then please consider doing sensitivity analyses using a different threshold in the definition of the outcome (e.g. 10% change instead of 20%), or argue why 20% was chosen. Please also report the frequency of the three categories of the outcome.

*We appreciate Reviewer 3 for raising this concern.* *We incorporated a 3-label outcome since it is not necessarily the case that an individual will regress to the population mean after enough time (e.g., a person who consistently spends no time in range may tend to stay out of range, or a person who consistently spends time in range may tend to stay within range). The “negligible” change label (+/- 20%) was meant to account for instances in the population-level model where someone is high and stays high, or is stable and stays stable. We believe the 20% cutoff is hyperparameter that different researchers could change for their specific tasks, and was chosen heuristically for the purposes of this work since we were not focused on maximizing performance. This work is meant to identify potential engineered covariates in a population-level model that could be incorporated into personalized/individual-level models in the future, but we believe individual-level models that are more intended for consumer use is a latter project that is out of scope of what we intended. We have added more to the discussion regarding how these features could be adapted to individual-level models in the future (lines 163-173). We have also included frequency of the three categories in Table 1 (line 140).*

3.c I was also wondering why there was focus on hyperglycemia and not hypo (below range).

*We thank Reviewer 3 for pointing out a source of ambiguity. We have added a point in the Discussion that the reason we focused on hyperglycemia is that it is more commonly associated with chronic mismanagement/mistreatment of blood-glucose concentration. Hypoglycemia is more associated with side-effects of blood-sugar-lowering medication (lines 187-189).*

3.d Based on the following sentence: ‘long-term predictions give insight into both behavioral and lifestyle changes that could reduce the risk of dysregulation in the first place’, it seems to me that the authors are interested in using the model to make individual recommendations to individuals. This is often done by using thresholds, e.g. if the probability is above 20% then do the following action (e.g. exercise)… In this case, discrimination is not the best performance metric to report. Calibration (e.g. calibration in the large and calibration slope, see BMJ 2024; 384 :e074819) would be more relevant, because that gives an idea of the magnitude of predicted probabilities vs actual outcomes.

*We appreciate Reviewer 3’s feedback. We have added calibration curves for the output probabilities for all three classes trained on the XGBoost model and have included them as a supplementary figure (Supplementary Figure 3).*

3.e Predictors
I am curious why only two days are used as predictors and why a longer period was not utilized. The interesting results described in Fig 1 are not really utilized in the prediction model as far as I understand. I suggest the authors include all predictors tested and how they were defined in a supplement. The top 20 are presented, but the names on Fig 4D are not all self-explanatory (e.g. YesterToday_SD2). Please include the definition of all of these. ‘If they provided predictive power then they would appear in the top 20 list’ Just because a predictor appears in the top 20, it doesn’t mean that it is relevant, if all the other tested predictors are irrelevant, so I suggest reformulating this. The authors then mention that 5 complexity features made it into the top 20, but I can only find CI_sleep that explicitly mentions CI. Which four am I missing?

*We appreciate Reviewer 3 for pointing out an area in the results we could have clarified. The results from Figure 1 are an example of across-day variance. While that was 14-day standard deviation at specific times of day, we instead used a corollary for the machine learning model inspired by non-linear dynamics which was more equipped for 2-day windows: short- and long-term variance from a Poincare plot. We have reworded the statement regarding additional predictive power, and have edited the “5 complexity features” to instead by “5 complexity and across-day variance features” (lines 129-134).*

**Minor**
3.f I suggest using ‘people with diabetes’ instead of DM subjects/diabetic individuals, or participants instead of subjects throughout the manuscript (Diabetes Care 2017;40(12):1790–1799).

*We appreciate Reviewer 3’s recommendation for the use of “people with diabetes” instead of “DM subjects/diabetic individuals”. We have gone through and updated the descriptions with the recommended terminology (lines 27, 33, 147, 190).*

3.g Fig 1C the unit of TIR is % I suspect and not hours.

*We appreciate Reviewer 3’s identification of the incorrectly labeled units for the y-axis label in Figure 1C. We have changed ‘hours’ to ‘%’ to reflect the correct units.*

3.h Have the authors considered separating weekends in the analysis and creating weekday- and weekend-specific features? People often have very different behavioral patterns in these two periods.

*We thank Reviewer 3 for recommending the separation of weekday and weekend features. We agree that such separation could yield interesting results if additional behavioral surveys or datastreams were available. However, given the scope of the novel feature engineering was on the 5-minute level data, we believe that the incorporation of day-of-week is more appropriate for a personalized or individual-level model.*

3.i It is not very reader-friendly to have the Methods section at the end of the manuscript as there are several places in the Results where the authors refer to it. If it’s journal style, then it is a comment rather to the editorial board, otherwise I suggest moving the Methods before the Results.

*We understand Reviewer 3’s comment; however, the PLOS Digital Health* *submission guidelines list the Methods section as following the Discussion section.*

3.j Please make the figures self-explanatory i.e. introduce all abbreviations again.

*We thank Reviewer 3 for their recommendation to reintroduce all abbreviations. We have gone through the captions and reintroduced terms where necessary.*

3.k Please improve the resolution of some figures (e.g. Fig 3).

*We thank Reviewer 3 for pointing out low dpi on some figures. Figures 2 and 3 have had their resolution increased.*

3.l Really nice with the PCA analysis and the figure. I suggest moving some of the labels (excellent with direct labeling) so that they are not overlapping.

*We thank Reviewer 3 for their appreciation of the PCA figure. We have cleaned up the text at the end of their respective vectors, and have only included features that have relationships to the principle components that are greater than or equal to a magnitude of 0.25 (to remove clutter).*

3.m I suspect there was more data available than 2 weeks per person. How were the 2 weeks chosen? Randomly?

*We appreciate Reviewer 3’s question regarding the selection of samples. We have added in clearer language in the* ***Methods*** *-* ***Variability of glucose concentrations at the same time of day (ToD) across multiple days reveals an increased risk of glycemic dysregulation*** *that the two-week windows were selected from the same time of year for all individuals based on the fact that all participants had CGM data during that time and had very little missing data per day (lines 255-256).*

3.n Why didn’t the authors tune the XGBoost model?

*We appreciate Reviewer 3’s question. One of the primary goals of the manuscript was to highlight the benefit of chronobiologically-informed feature engineering by extracting informative covariates about the target variable that are independent of the typical statistical features (central tendency, variance, skew, etc.) extracted from longitudinal timeseries. To this end, the XGBoost model was more of a validation of the addition of these novel features for the feasibility of predicting longer-term glycemic control. In the future, if a pure machine learning focused project were to be developed, hyperparameter optimization would be an ideal option for maximizing the performance of a model that may be placed in production. We have added a brief summary of this to the Discussion (lines 163-167)*

Again, we want to thank the reviewers for their thoughtful insight. We believe this process has made the manuscript stronger and more impactful as a whole. We also thank the Editor for the opportunity to improve this publication. Please contact Benjamin Smarr (bsmarr@ucsd.edu) if additional edits are required or if any further information regarding this manuscript would be helpful.

Sincerely,

Benjamin L. Smarr

Assistant Professor

Department of Bioengineering and Halicioğlu Data Science Institute

University of California, San Diego
