# Supplementary material for: Chronobiologically-informed features from CGM data provide unique information for XGBoost prediction of longer-term glycemic dysregulation in 8,000 individuals with type-2 diabetes
Source: PLOS Digit Health. 2025 Apr 9;4(4):e0000815. doi: 10.1371/journal.pdig.0000815 (PMC11981153; doi:10.1371/journal.pdig.0000815)
Supplement: Attachment — Submitted filename: Response to Reviewers_R2.docx [file pdig.0000815.s008.docx]

HALICIOĞLU DATA SCIENCE INSTITUTE

9500 GILMAN DRIVE

LA JOLLA, CALIFORNIA

92093-0555

BENJAMIN L. SMARR

ASST. PROF. BIOENGINEERING AND HDSI

February 26, 2025

Dear Dr. Leo Anthony Celi,

Thank you for providing review comments for our manuscript *Chronobiologically-Informed Features from CGM Data Provide Unique Information for XGBoost Prediction of Longer-Term Glycemic Dysregulation in 8,000 Individuals with Type-2 Diabetes*. We appreciate the most recent comments by the reviewers to improve the manuscript.

We provide responses item by item below with line numbers as present in the ‘Revised Manuscript with Track Changes’ file.

*Additional Editor Comments***:**

Thank you for the clarification. Upon reviewing the response again, I see that the issue regarding data availability has not been fully addressed. As per PLOS’s data availability policy, all underlying data must be made publicly available prior to acceptance, not just post-acceptance. This means that the data should either be included as part of the manuscript submission or deposited in a public repository with unrestricted access at the time of submission. Once this is resolved, I can proceed with the next steps in the review process.

*We thank the editor for working with us through this process to understand the data storage requirements associated with PLOS’s data availability policy. The data and associated code are now stored in the UC San Diego Library’s Open Data Repository for reproducibility and potential future analyses (*[*https://doi.org/10.6075/J0BR8SK9*](https://doi.org/10.6075/J0BR8SK9)*).*

*Reviewers' Comments***:**

*Review Comments to the Author*

*Reviewer #2*: The authors have responded to my comments, carefully. No further suggestions. I think that the paper can be published as it is without any other request.

*We appreciate Reviewer 2’s feedback and are pleased that they approve of the current version of the manuscript.*

*Reviewer #3*: The authors have conducted a thorough revision, which improved the quality of the paper. I have two remaining comments.

3a. I find the new supplementary figures very informative, but they are not mentioned in the manuscript, so readers' attention is not called to them. I suggest to include them, especially the one on calibration, because the new features seem to improve this aspect.

*We thank Reviewer 3 for this feedback. We have added in mentions to the supplementary material in the manuscript for Supplementary Figure 1 – mCI Examples (line 96), Supplementary Figure 2 – Calibration Curves (line 128), and another reference to Supplementary Figure 1 in the methods describing the mCI algorithm (lines 277-278)*

3b. The authors state that both (some) de-identified data and code are available in the University of California, San Diego’s Open Data Repository. Please provide links (or doi) to help readers navigate to this useful resource.

*The data and associated code are now stored in the UC San Diego Library’s Open Data Repository for reproducibility and potential future analyses (*[*https://doi.org/10.6075/J0BR8SK9*](https://doi.org/10.6075/J0BR8SK9)*). This data set is cited and directly linked/referenced in the Data Availability statement (line 306).*

Again, we want to thank the reviewers for their recommended improvements. We believe this process has made the manuscript stronger and more impactful as a whole. We also thank the Editor for the opportunity to improve this publication. Please contact Benjamin Smarr (bsmarr@ucsd.edu) if additional edits are required or if any further information regarding this manuscript would be helpful.

Sincerely,

Benjamin L. Smarr

Assistant Professor

Department of Bioengineering and Halicioğlu Data Science Institute

University of California, San Diego
